# Supplementary material for: Clinical Significance of Tumour-Infiltrating B Lymphocytes (TIL-Bs) in Breast Cancer: A Systematic Literature Review
Source: Cancers (Basel). 2023 Feb 11;15(4):1164. doi: 10.3390/cancers15041164 (PMC9953777; doi:10.3390/cancers15041164)
Supplement: Supplementary file 1 [file cancers-15-01164-s001.zip › Table S1.pdf]

Table S1 - Prognostic and predictive significance of TIL-Bs in breast cancer

Year	Authors	Origin of cohorts	Number of patients	Breast cancer type	Neoadjuvant treatment given	TIL-B evaluation method(s) on baseline samples	Full-face sections vs Tissue microarray (TMA)	Median follow-up (months)	Significantly prognostic and/or predictive	Details of clinical significance
2021	Kuroda et al.[30]	Japan	114	TNBC	Not specified	IHC (CD20, CD38, CD138)	Full-face sections	35 for RFS, 42.5 for OS	Yes, prognostic (positively)	↑iCD20+ TILs: ↑RFS (p=0.025) & ↑OS (p=0.026)<br>↑sCD20+ TILs: ↑RFS (p= 0.006) & ↑OS (p=0.004)<br>↑sCD38+ TILs: ↑RFS (p=0.008) & ↑OS (p=0.011)
2019	Garaud et al.[31]	Belgium	249	136 HER2+<br>113 TNBC	Not specified	IHC (CD20)	Full-face sections	120	Yes, prognostic (positively)	TILB+: ↑DFS (p=0.03) & ↑OS in HER2+ (p=0.04);<br>TILB+:↑DFS (p=0.02) & ↑OS in TNBC (p=0.01)
2018	Yeong et al.[32]	Singapore	269	TNBC	Not specified	IHC (CD20, CD38),	TMA	97	Yes, prognostic (positively)	↑iCD20+ TILs: ↑DFS (p=0.046) & ↑OS (p=0.042)<br>↑iCD38+ TILs: ↑DFS (p=0.007) & ↑OS (p=0.020)
2018	Xu et al.[33]	China	102	91 TNBC<br>11 non-TNBC	No	IHC (CD20)	Full-face sections	60	Yes, prognostic (positively)	↑CD20+ TILs: ↑OS (p=0.04)
2018	Arias-Pulido et al.[34]	Algeria	221	44 TNBC<br>177 non-TNBC	Yes (CT 95%, HT 64%, RT 84%)	IHC (CD20)	TMA	96	Yes, prognostic and predictive (positively)	↑CD20+ TILs:: ↑DFS (p=0.046) & ↑pCR (p=0.003)<br>↑CD20+ TILs/PD-1+ TILs: ↑pCR (p=0.04)<br>↑CD20+ TILs/PD-L1+ tumour cells: ↑pCR (p= 0.005)
2016	Song et al.[35]	Korea	108	TNBC	Yes (CT 100%)	IHC (CD20),	Full-face sections	34.9	Yes, prognostic and predictive (positively)	↑CD20+ TILs: ↑DFS (p = 0.002) & ↑pCR (p=0.037)
2016	Miligy et al.[36]	UK	80	36 DCIS<br>44 DCIS + IDC	No	IHC (CD19, CD20, CD138)	Full-face sections	266	Yes, prognostic (negatively)	↓Peri-Tu TIL-Bs: ↑RFS (p=0.008)<br>↓Para-Tu TIL-Bs: ↑RFS (p=0.04)

Table S1 - Prognostic and predictive significance of TIL-Bs in breast cancer

Year	Authors	Origin of cohorts	Number of patients	Breast cancer type	Neoadjuvant treatment given	TIL-B evaluation method(s) on baseline samples	Full-face sections vs Tissue microarray (TMA)	Median follow-up (months)	Significantly prognostic and/or predictive	Details of clinical significance
2014	Garcia-Martinez et al.[37]	Spain	121	13 HER2+<br>26 TNBC<br>77 ER/PR+<br>5 unclassifiable	Yes (CT)	IHC (CD20)	TMA	60	Yes, predictive (positively)	↑CD20+ TILs: ↑pCR ($p=0.005$)
2014	Brown et al.[38]	USA	95	Invasive BC, including TNBC (number not specified)	Yes (CT)	IF (CD20)	Full-face sections	NA	Yes, predictive (positively)	↑CD20+ TILs: ↑pCR ($p=0.019$)
2013	Mohammed et al.[39]	UK	338	IDC (number of intrinsic subtypes not specified)	No	IHC (CD20, CD138)	TMA (40 also assessed with full-face sections	164	Yes, prognostic (negatively)	↑CD138+ TILs: ↓BCSS ($p<0.001$)
2012	Mohammed et al.[40]	UK	468	IDC (number of intrinsic subtypes not specified)	No	Histology	Full-face sections	165	Yes, prognostic (negatively)	↑plasma cells: ↓BCSS ($p<0.001$)
2012	Mahmoud et al.[41]	UK	1470	288 TNBC<br>1078 non-TNBC<br><br>Histology:<br>75% IDC<br>7% lobular<br>3% tubular<br>12.5% others	No	IHC (CD20)	TMA	128	Yes, prognostic (positively)	↑total CD20+: ↑BCSS ($p=0.025$) & ↑DFI ($p=0.002$)<br><br>↑iCD20+: ↑BCSS ($p=0.005$) & ↑DFI (details not shown)<br><br>↑asCD20+: ↑BCSS ($p=0.032$)<br><br>↑dsCD20+: ↑BCSS ($p=0.005$) & ↑DFI (details not shown)
2012	Eiro et al.[42]	Spain	102	Early invasive BC (including 33 with basal-like phenotype)	No	IHC (CD20)	TMA	60	No, not prognostic	CD20+ cells showed no significant associations with RFS (details not shown)

Table S1 - Prognostic and predictive significance of TIL-Bs in breast cancer

Year	Authors	Origin of cohorts	Number of patients	Breast cancer type	Neoadjuvant treatment given	TIL-B evaluation method(s) on baseline samples	Full-face sections vs Tissue microarray (TMA)	Median follow-up (months)	Significantly prognostic and/or predictive	Details of clinical significance
2011	West et al.[43]	Canada	113 NACT cohort<br>255 ACT cohort	ER-negative BC	Yes (CT)	IHC (CD20)	TMA	83 (ACT cohort)	No, not prognostic nor predictive	CD20+ cells failed to predict pCR in NACT cohort (details not shown)<br><br>CD20-high and CD20-low patients did not show statistically significant difference in survival following ACT (details not shown)

TNBC, triple negative breast cancer; IHC, immunohistochemistry; RFS, relapse-free survival; OS, overall survival; iCD20+ TILs, intratumoural CD20+ TILs; sCD20+ TILs, stromal CD20+ TILs; sCD38+ TILs, stromal CD38+ TILs; HER2+, human epidermal growth factor receptor-2 positive; DFS, disease-free survival; CT, chemotherapy; HT, hormonal therapy; RT, radiotherapy; pCR, pathological complete response; DCIS, ductal carcinoma in-situ; IDC, invasive ductal carcinoma; Peri-Tu TIL-Bs, peritumoural B lymphocytes; Para-Tu TIL-Bs, paratumoural B lymphocytes; ER/PR+, estrogen receptor and/or progesterone receptor positive; BC, breast cancer; IF, immunofluorescence; BCSS, breast cancer-specific survival; DFI, disease-free survival; asCD20+, adjacent stromal CD20+ cells; dsCD20+, distant stromal CD20+ cells; NACT, neoadjuvant chemotherapy; ACT, adjuvant chemotherapy
