# Supplementary material for: Clinical Significance of Tumour-Infiltrating B Lymphocytes (TIL-Bs) in Breast Cancer: A Systematic Literature Review
Source: Cancers (Basel). 2023 Feb 11;15(4):1164. doi: 10.3390/cancers15041164 (PMC9953777; doi:10.3390/cancers15041164)
Supplement: Supplementary file 1 [file cancers-15-01164-s001.zip › Table S2.pdf]

Table S2 – Summary of TIL-Bs scoring methodology

Year	Authors	Antibody clone and producer	Compartment(s) of TIL-Bs specified: intratumoural vs stromal	Details of TIL-Bs compartmentalization	Phenotypic subtyping of TIL-Bs	Details of subtypes	Categorization of abundance	Cut-off value
2021	Kuroda et al.[30]	CD20: L26, Nichirei<br>CD38: SPC32, Novocastra (Leica)<br>CD138: MI15, Novocastra (Leica)	✓	1) Intratumoural: in direct contact with tumour cells with no intervening stroma<br>2) Stromal: within the tumour stroma and not directly infiltrating tumour cells	✓	1) iCD20+<br>2) iCD38+<br>3) iCD138+<br>4) sCD20+<br>5) sCD38+<br>6) sCD138+	High vs Low	iCD20+: 1<br>iCD38+: 4<br>iCD138+: 10.5<br>sCD20+: 61<br>sCD38+: 39.5<br>sCD138+: 35<br>(number of positive cells)
2019	Garaud et al.[31]	CD20: L26, Agilent	✗	NA	✗	NA	Positive vs Negative	For HER2+: 5.5%<br>For TNBC: 2.75%<br>(percentage of total CD45+ cells)
2018	Yeong et al.[32]	CD20: L26, Dako<br>CD38: SPC32, Novocastra	✓	1) Intratumoural<br>2) Stromal	✓	1)iCD20+<br>2)iCD38+<br>3)sCD20+<br>4)sCD38+	High vs Low	iCD20+: 5%<br>iCD38+: 5%<br>sCD20+: 1%<br>sCD38+: 1%<br>(percentage of intratumoural or stromal areas occupied by labelled cells)
2018	Xu et al.[33]	CD20: Dako	✓ (in intratumoural stromal except those inside lymph vessels)	NA	✗	NA	High vs Low	115<br>(mean number of positive cells in 3 different areas that had the most stained cells in the intratumoural stroma at 400 X magnification)
2018	Arias-Pulido et al.[34]	CD20: L26, Dako	✓ (in tumour stroma, as per recommendations by the International TILs Working Group 2014)	NA	✗	NA	Positive vs Negative	1%<br>(percentage of membranous CD20 staining in TILs)

Table S2 – **Summary of TIL-Bs scoring methodology**

Year	Authors	Antibody clone and producer	Compartment(s) of TIL-Bs specified: intratumoural vs stromal	Details of TIL-Bs compartmentalization	Phenotypic subtyping of TIL-Bs	Details of subtypes	Categorization of abundance	Cut-off value
2016	Song et al.[35]	CD20: Novocastra	✗ (in tumour area)	NA	✗	NA	Continuous variable: Number of CD20+ lymphocytes in the tumour area divided by tumour area)	NA
2016	Miligy et al.[36]	CD19: LE-CD19, Dako<br>CD20: L26, Dako<br>CD138: MI15, Dako	✓	1) Intratumoural: within DCIS or invasive component<br>2) Peritumoural: < 0.5 mm from DCIS margin or within one tumour cell<br>diameter of invasive component<br>3) Paratumoural: > 0.5 mm and up to 1 mm from DCIS margin<br>4) Stromal: > 1 mm and up to 2 mm from DCIS margin or in stroma more than one tumour cell diameter away from invasive component	✓	1) Intratumoural TIL-Bs<br>2) Peritumoural TIL-Bs<br>3) Paratumoural TIL-Bs<br>4) Stromal TIL-Bs<br>(TIL-Bs: CD19+ and/or CD20+ cells)<br><br>5) Stromal plasma cells (CD138+ cells)	High vs Low	Numerical value not shown (Percentage of intratumoral or stromal area in hotspot occupied by stained cells at 400X magnification)
2014	Garcia-Martinez et al.[37]	CD20: Dako	✗ (in tumour area)	NA	✗	NA	Continuous variable: CD20+ cell count/mm^2	NA
2014	Brown et al.[38]	CD20: L26, Dako	✓ (in tumour stroma)	NA	✗	NA	High vs Low	59<br>(CD20 stromal AQUA score)
2013	Mohammed et al.[39]	CD20: L26, Dako<br>CD138: MI15, Dako	✗ (at the invasive margin)	NA	✓	CD20+ TIL-Bs<br>CD138+ TIL-Bs	Low vs Moderate vs High	CD20+ CD138+<br>Low: 0 0<br>Moderate: 1-12 1-9<br>High: ≥13 ≥ 10<br>(mean percentage of stained cell type in a minimum of 4 to a maximum of 12 fields at 400 X magnification)

Table S2 – Summary of TIL-Bs scoring methodology

Year	Authors	Antibody clone and producer	Compartment(s) of TIL-Bs specified: intratumoural vs stromal	Details of TIL-Bs compartmentalization	Phenotypic subtyping of TIL-Bs	Details of subtypes	Categorization of abundance	Cut-off value
2012	Mohammed et al.[40]	NA (by morphology alone)	✗ (at the invasive margin)	NA	✓	Plasma cells	Low vs High	Low: <3<br>High: ≥3<br>(mean percentage of stained cell type in a minimum of 4 to a maximum of 12 fields at 400 X magnification)
2012	Mahmoud et al.[41]	CD20: L26, Dako	✓	1) Intratumoural: within tumour nests<br>2) In adjacent stroma: within one tumour cell diameter of the tumour<br>3) In distant stroma: more than one tumour cell diameter away from the tumour	✓	1) Total CD20+<br>2) Intratumoural CD20+<br>3) Distant stromal CD20+<br>4) Adjacent stromal CD20+	High/Positive vs Low/Negative	1) Total CD20+: 5<br>2) Intratumoral CD20+: 1<br>3) Adjacent stromal: 1<br>4) Distant stromal: 5
2012	Eiro et al.[42]	CD20: Dako	✗ (at the invasive front)	NA	✗	NA	> median vs<br>≤ median	29<br>(Median number of CD20+ cells in 1 mm^2 area of the invasive front, evaluated in 5 fields at 400 X magnification)
2011	West et al.[43]	CD20: polyclonal, catalogue no. RB-9013, Lab Vision	✓ (intraepithelial: within tumour cell nests or in direct contact with tumour cells)	NA	✗	NA	> median (high) vs<br>≤ median (low)	Numerical value not shown<br>(Number of intraepithelial CD20+ lymphocytes within an area of 0.56 mm^2, divided by the fraction of grid area occupied by tumour epithelium)

AQUA, automated quantitative analysis
